# *Vive la différence*: naming structural variants in the human reference genome

**DOI:** 10.1186/1479-7364-7-12

**Published:** 2013-05-01

**Authors:** Ruth L Seal, Mathew W Wright, Kristian A Gray, Elspeth A Bruford

**Affiliations:** 1 HUGO Gene Nomenclature Committee (HGNC), European Bioinformatics Institute (EMBL-EBI), Wellcome Trust Genome Campus, Hinxton, Cambridgeshire CB10 1SD, UK

**Keywords:** Gene nomenclature, Reference genome, Structural variants, Human

## Abstract

The HUGO Gene Nomenclature Committee has approved gene symbols for the majority of protein-coding genes on the human reference genome. To adequately represent regions of complex structural variation, the Genome Reference Consortium now includes alternative representations of some of these regions as part of the reference genome. Here, we describe examples of how we name novel genes in these regions and how this nomenclature is displayed on our website, http://genenames.org.

## Letter to the Editor

The HUGO Gene Nomenclature Committee (HGNC) [1] is the only resource with the authority to name human genes. Our philosophy has always been to assign symbols to genes to enable communication and for this nomenclature to evolve as required with new technology and discoveries. Since the initial release of the human genome sequence, we have worked closely with annotators and researchers towards naming all the genes from this source. Historically, we have also approved symbols for genes that are not part of the reference genome where particular communities have requested this. Examples include the naming of structural variants within the human leukocyte antigen (HLA; major histocompatibility complex) and killer-cell immunoglobulin-like receptor (KIR) gene families, both of which have dedicated nomenclature committees [2,3] that work directly with the HGNC, providing us with further confidence in the existence of these genes.

Since 2009, the human reference genome assembly has been maintained by the Genome Reference Consortium (GRC), a body of experts in genome assembly and annotation [4]. As part of the human reference genome, the GRC includes representations of common structural variation in the form of separate assembly units, which they call *novel patches* upon release and then label *alternate loci* when they are subsequently incorporated into the next version of the human reference genome. The sequence for these regions is submitted by experts in the field and then annotated by members of the GRC. Due to the difficulties in annotating regions of variation consistently and reliably, we have chosen to restrict our future naming of structural variants to those annotated by the GRC.

The *alternate loci* in the current GRC human reference genome version, GRCh37, encompass most of the structural variants that we had previously named. This includes *alternate loci* for seven major histocompatibility complex (MHC) haplotypes (see Figure 1 in [4]), which were previously sequenced and annotated by the MHC Haplotype Consortium [5]. Their inclusion into the reference means that each haplotype has its own assembly unit with a unique sequence accession that contains anchor sequences to allow the correct placement of the *alternate locus* onto 6p21.3. The haplotypes contain several named HLA genes (*HLA-DRB3*, *HLA-DRB4*, *HLA-DRB2*, *HLA-DRB7* and *HLA-DRB8*) that were not included in previous genome versions. It should be noted that these haplotypes also carry other named genes that were present in previous human genome assemblies, such as *HLA-C*. The same gene symbol will always be used to refer to a gene whether it is on the reference-assembled chromosomes or on a variant haplotype.

**Figure 1:**
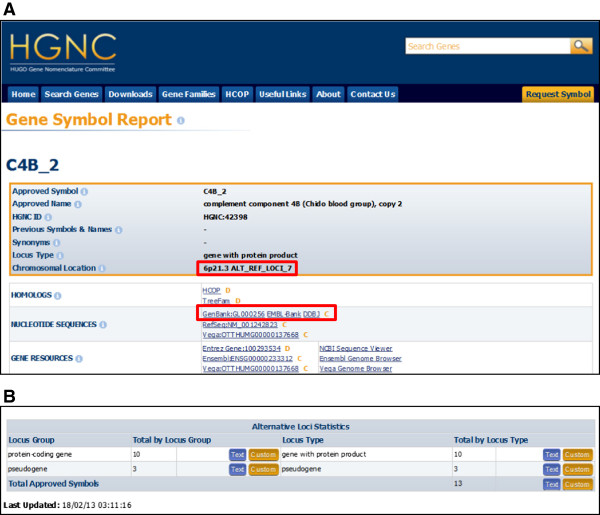
**Finding gene nomenclature for structural variants on the HGNC website.** (**A**) This shows the top of the Gene Symbol Report for the *C4B_2* gene. Users can see that this gene is annotated on an *alternate locus* due to the two fields indicated with *red boxes*. The ‘Chromosomal Location’ field shows the assembly unit name for the *alternate locus* on which *C4B_2* is located, while the ‘NUCLEOTIDE SEQUENCES’ field contains the sequence accession for the assembly unit; clicking on the links will take users to the sequence accession record. (**B**) Users can view and download statistics for named genes only found on variant assembly units using our ‘Alternative Loci Statistics’ table. They can choose simply to download the approved symbols, names and related information using the ‘Text’ button or click the ‘Custom’ button to be taken to our Custom Downloads tool, which allows users to select the exact data fields that they wish to download.

The MHC haplotype assembly unit ALT_REF_LOCI_7 [GenBank:GL000256] carries a novel extra copy of the *C4B* gene. We have now named this extra copy *C4B_2* for ‘complement component 4B (Chido blood group), copy 2’ to reflect its relationship to the single-copy gene on the main reference assembly, *C4B*, ‘complement component 4B (Chido blood group)’. We have reserved the underscore ‘_’ symbol to be used only in symbols for genes on structurally variant regions. To make it clear to the users of our database, when genes are only annotated on variant assembly units, we display the assembly unit name next to the chromosomal location and the assembly unit sequence accession ID is listed within the Gene Symbol Report (Figure 1A).

Structural variants do not have to correspond to the entire haplotypes to be included in the genome reference and may represent smaller structurally complex regions. Previously, the GRC contacted us to request a symbol for a small genomic deletion on chromosome 22 that is currently annotated on a *novel patch*. This common functional variant is the result of a deletion between the fifth exon of *APOBEC3A* and the eighth exon of *APOBEC3B*[6]. As the deletion affects both the *APOBEC3A* and *APOBEC3B* genes, it cannot be considered as an allele of either gene, and since it is found in over 20% of the worldwide population, we decided that it would be useful for the research community if we assigned it a specific gene symbol. We have named this variant *APOBEC3A_B* for “APOBEC3A and APOBEC3B deletion hybrid”, and we include the chromosomal location “22q13 GRCh37 novel patch” and the sequence accession [GenBank:GL383583] for the patch in the Gene Symbol Report. The *novel patch* will be incorporated into the human reference assembly as part of the next release, GRCh38, and we will update the chromosomal location to include the new assembly unit name when this occurs. The GRC has also included eight *novel patches* for the leukocyte receptor complex, meaning that most of the KIR genes that we had previously named now have representation in the reference genome. Only *KIR2DL5B* and *KIR2DS3* are still not represented; therefore, we have displayed their chromosomal location as “19q13.4 unplaced”.

We have also created a separate table called Alternative Loci Statistics on our Statistics and Downloads page [7] so that our users may easily view and download data on all named genes that are only found on *alternate loci* (Figure 1B). We will continue to work with the GRC to provide nomenclature for novel genes annotated on *alternate loci* and *novel patches*. If you have any queries, please contact us at hgnc@genenames.org, and to follow updates on our project, please subscribe to our newsletter using our feedback form [8].

## Competing interests

The authors declare that they have no competing interests.

## Authors’ contributions

RLS drafted the manuscript and, along with EAB and MWW, formulated the nomenclature for structural variants. KAG implemented the display of structural variants on the HGNC website. All authors read and approved the final manuscript.
